# Growth Hormone and Neuronal Hemoglobin in the Brain—Roles in Neuroprotection and Neurodegenerative Diseases

**DOI:** 10.3389/fendo.2020.606089

**Published:** 2021-01-08

**Authors:** Marion Walser, Johan Svensson, Lars Karlsson, Reza Motalleb, Maria Åberg, H Georg Kuhn, Jörgen Isgaard, N David Åberg

**Affiliations:** ^1^ Department of Internal Medicine, Institute of Medicine, The Sahlgrenska Academy, University of Gothenburg, Gothenburg, Sweden; ^2^ Region Västra Götaland, Sahlgrenska University Hospital, Gothenburg, Sweden; ^3^ Department of Clinical Neuroscience, Institute of Neuroscience and Physiology, The Sahlgrenska Academy at University of Gothenburg, Gothenburg, Sweden; ^4^ The Queen Silvia Children’s Hospital, Sahlgrenska University Hospital, Gothenburg, Sweden; ^5^ School of Public Health and Community Medicine at University of Gothenburg, Gothenburg, Sweden; ^6^ Institute for Public Health, Charité – Universitätsmedizin Berlin, Berlin, Germany; ^7^ Hunter Medical Research Institute, University of Newcastle, Newcastle, NSW, Australia

**Keywords:** hemoglobin, growth hormone, insulin-like growth factor I, anemia, erythropoietin, ischemia, stroke, neurodegenerative diseases

## Abstract

In recent years, evidence for hemoglobin (Hb) synthesis in both animal and human brains has been accumulating. While circulating Hb originating from cerebral hemorrhage or other conditions is toxic, there is also substantial production of neuronal Hb, which is influenced by conditions such as ischemia and regulated by growth hormone (GH), insulin-like growth factor-I (IGF-I), and other growth factors. In this review, we discuss the possible functions of circulating and brain Hb, mainly the neuronal form, with respect to the neuroprotective activities of GH and IGF-I against ischemia and neurodegenerative diseases. The molecular pathways that link Hb to the GH/IGF-I system are also reviewed, although the limited number of reports on this topic suggests a need for further studies. In summary, GH and/or IGF-I appear to be significant determinants of systemic and local brain Hb concentrations through mediating responses to oxygen and metabolic demand, as part of the neuroprotective effects exerted by GH and IGF-I. The nature and quantity of the latter deserve further exploration in specific experiments.

## Introduction

**Figure 1:**
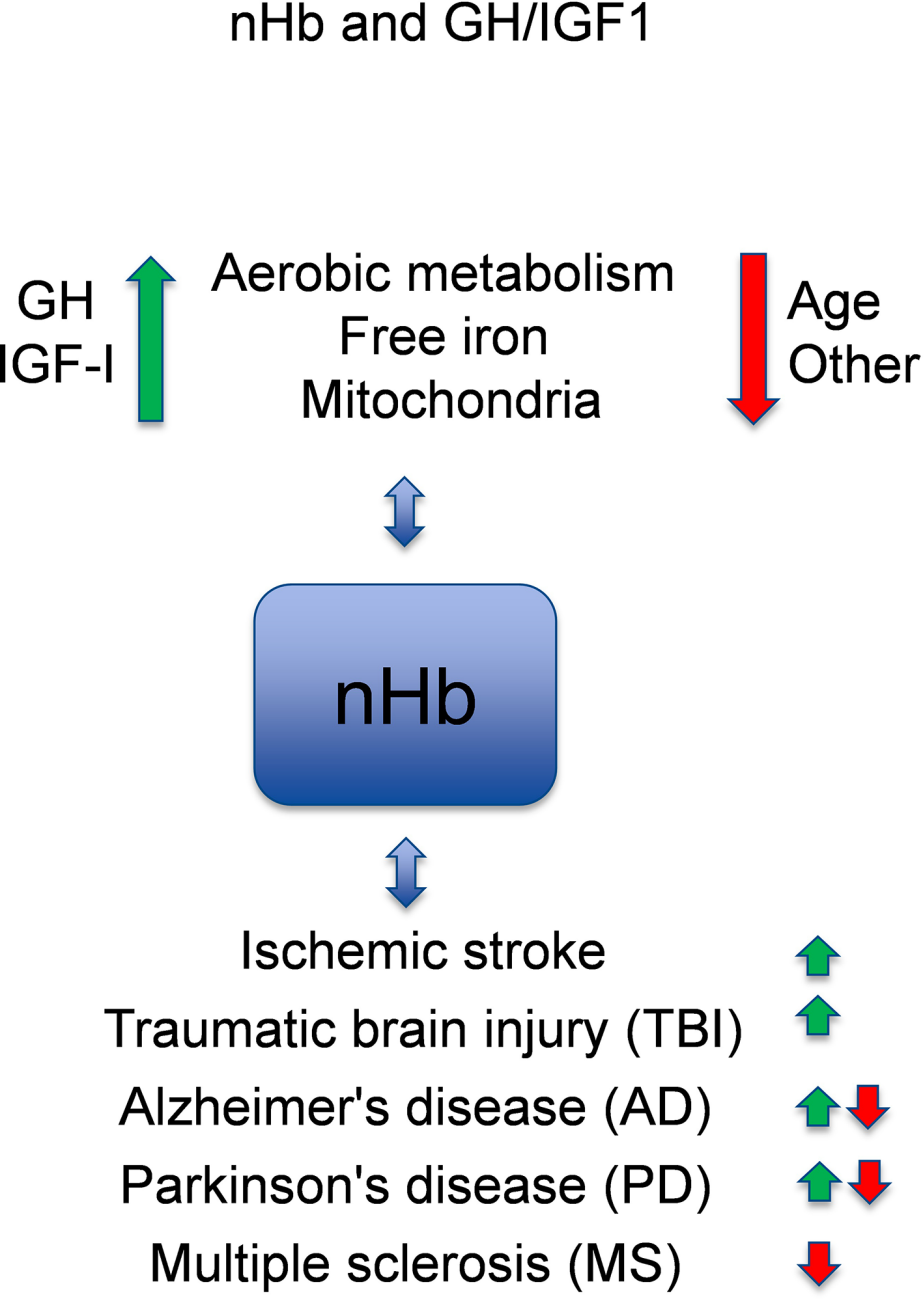
Model of crosstalk between neuronal hemoglobin (nHb), growth hormone (GH), and insulin-like growth factor 1 (IGF-I), and the known relations to brain diseases. With respect to interactions with neuronal Hb (nHb), the green arrows are beneficial, and the red arrows are detrimental pathways. Please, observe that circulating or free HbA has other effects not included in this figure. In general, GH and IGF-I levels within the normal ranges are optimal for the metabolism of oxygen and iron and for the mitochondria, which is beneficial for the brain. With age or for other reasons (e.g., genes, environment), the levels of GH and IGF-I decline, and the metabolism deteriorates leading to lower levels of nHb. This has adverse effects on degenerative brain diseases (AD, PD, MS), although in some aspects nHb may also respond in a manner that slows the disease progression. References for the depicted relationships are given in the main text.

It is known that GH and IGF-I are associated with circulating hemoglobin (Hb) levels in health and disease states [for reviews, see (1, 2)]. Recently, it has been discovered that Hb is also locally expressed in the brain (3, 4) [for a review, see (5)], which suggests that the oxygen-binding nature of hemoglobin may have a neuroprotective role in the brain. Although each of these topics have been reviewed previously, a comprehensive review of circulating and local Hb in relation to the neuroprotective and cognitive effects of GH and IGF-I has to the best of our knowledge not been presented. Here, we present an updated review of the potential links between these areas of neuroscience, in which we also identify knowledge gaps that warrant further investigation (**Figure 1**).

### Hemoglobin Biochemistry and Relationship to Other Globins

Hemoglobins are widespread in the biosphere and are found in all kingdoms of organisms, including prokaryotes, fungi, plants, and animals. The ancestral Hb gene existed phylogenetically before the divergence of plants and animals (6). There are at least four different globins in vertebrates (**Table 1**): Hb, myoglobin (Mb), cytoglobin (Cygb), and neuroglobin (Ngb) [for reviews, see (5, 9)]. With respect to the brain, it is important to note that neuronal Hb (nHb) is present in parallel with the structurally different Ngb, with which it shares 25% protein sequence identity (10). Although the presence of both globins has been demonstrated (11), their relative amounts and the overlaps in their expression profiles are only partly known (11). Unlike Hb, Ngb is not responsive to erythropoietin (EPO). Nevertheless, Ngb appears to have similar neuroprotective functionalities, as it has been shown to correlate with serum IGF-I in hypoxic-ischemic conditions (12). Mammalian Hb is a tetramer of 574 amino acids, consisting of two α-globin (Hba; 141 amino acids) subunits and two β-globin (Hbb; 146 amino acids) subunits. Each of these subunits is bound to a heme group (13), ensuring four oxygen-binding groups per tetramer. The tetramer forms HbA (hemoglobin A) in red blood cells and with a different name nHb in the brain, however identical in structure. The heme group contains four pyrrole rings, the nitrogen atoms of which coordinate a central iron atom that binds weakly to oxygen (14). The main function of Hb is to transport oxygen from the lungs to other tissues in the body in exchange for carbon dioxide, which is transported back to the lungs. Molecular oxygen, O_2_, is essential for cellular respiration in aerobic organisms. Aerobic respiration takes place in the mitochondria of cells and requires oxygen to create adenosine triphosphate (ATP), which provides the energy to drive numerous processes in living cells. However, Hb also functions as a redox enzyme, owing to its ability to bind to hydrogen peroxide (H_2_O_2_) (15). In addition, nHb may play a protective role against oxidative and nitrosative stresses by binding nitric oxide (NO), the strongest known ligand of the ferrous heme iron of Hb, with approximately 500,000-times higher affinity than oxygen (16, 17). Although the brain constitutes only ≈2% of bodyweight, it consumes ≈20% of the oxygen in the resting body (18), demonstrating the high energy demand of the brain. Therefore, it is no surprise that Hb is expressed locally in the brain, and it has been attributed numerous functions therein.

**Table 1:** Cell type-specific expression of vertebrate globins in the body and in the brain.

Hemoglobin tetramer	nHb/HbA				
Type of globin	hemoglobin α	hemoglobin β	myoglobin	cytoglobin	neuroglobin
Protein abbreviation	HBA	HBB	MB	CYGB	NGB
Transcript abbreviation used in Ms	Hba	Hbb	Mb	Cygb	Ngb
					
Identity vs Hbb	42%	100%	30%	33%	26%
Identity vs Ngb	30%	26%	25%	30%	100%
					
Cell type expression in body			cardiac myocytes	cardiac myocytes	sympathetic nerves
				smooth muscle cells	
			skeletal muscles	skeletal muscles	
					
Relative expression in tissues	neurons	neurons	N/A	neurons	neurons
					
red blood cells	186,1	523,2			
heart muscles			366,6	43,4	
skeletal muscles			871,2	17,1	
smooth muscle				17,6	
					
Olfactory region	5,8	10,8	N/A	5,5	6,4
Cerebral cortex	10	10,7	0,1	11,6	14,2
Hippocampal formation	5,2	17,9	0,2	4,2	1,7
Amygdala	7,1	20,9	0,2	6,2	4,2
Basal ganglia	6,6	21	0,2	8,3	4,8
Thalamus	2,2	3,8	N/A	2,2	0,9
Hypothalamus	5,1	17,4	0,1	11,2	34,5
Midbrain	10,1	31,6	0,2	6,3	2
Pons and medulla	3,3	23,7	N/A	17,7	8
Cerebellum	7,9	6,5	0,1	10,9	1,2
Corpus callosum	3	7,2	N/A	1,5	0,5
Spinal cord	7,9	26,5	0,2	3,5	1,4

References for these relationships are given in main text (section on Hemoglobin biochemistry and relation to other globins). For RNA normalized expression (NX) see https://www.proteinatlas.org/, and Proteomics. Tissue-based map of the human proteome (7). For homology percentages see (8), https://www.ebi.ac.uk/Tools/psa/emboss_matcher/ and https://www.uniprot.org/.

### Local Expression of Hemoglobin in the Brain

The presence of Hb-specific transcripts in the brain is a matter of controversy, as contamination by blood may cause false detection of brain Hb expression. Essentially, residual red blood cells in the brain could account for the detection of Hb mRNAs or proteins, which could be erroneously considered to be of parenchymal (non-erythroid) origin, especially in tissues where the capillaries are damaged and red blood cells have dispersed into the parenchyma. This is especially problematic in the case of Hb proteins in the brain, which probably should be attributed mostly to red blood cells. However, since circulating mature erythrocytes do not express mRNA transcripts after the reticulocyte state and the fact that reticulocytes constitute only about 1% of red blood cells, even in non-perfused brains, the presence of Hb transcripts in brain homogenates suggests that they are of parenchymal origin (19). The methodologic problem of red blood cell contamination is practically eliminated in studies of saline-perfused brains, resulting in minimal amounts of residual red blood cells. Further on, histologic techniques have shown specific neuronal and other non-erythrocyte expression (4, 20), although the relative amounts of circulating and local Hb in the living brain have to our knowledge not been studied. If local brain nHb constitutes even only 1–5% of the total Hb in the living brain, it could also have relevance for the hemodynamic response in functional imaging studies, which rely on the measurement of oxy- and deoxy-Hb concentrations either indirectly (fMRI-BOLD-signal) or directly (fNIRS-signal) (21).

**Table 1** provides a summary of the central nervous system (CNS) expression of endogenous CNS (non-erythrocyte) Hb. Hb has been found in the rodent and human brains (4, 20) and is expressed in neurons of both young and adult brains (3). Specifically, Hb is expressed in dopaminergic (DA) neurons, and to some degree in cortical and hippocampal astrocytes and mature oligodendrocytes (22). Moreover, *in vivo*, the expression of the neuronal Hba and Hbb proteins is colocalized in mouse dopaminergic neurons, further indicating that in most cases, the tetrameric Hb structure is intact in the brain. Thus, nHb probably exerts biochemical activities and biological functions similar to those associated with its roles in erythroid cells (23). This has been demonstrated by transfection of a murine DA cell line (MN9D) with α- and β-globin chains, which suggested that Hb expressed in the brain acts as a functional oxygen reservoir in anoxic and hypoxic conditions (22). Furthermore, in cortical pyramidal neurons, Hbb expression has been found to interact with mitochondrial proteins such as ATP synthase and ADP/ATP translocase (24). These interactions between Hbb and mitochondrial components suggest that Hbb is associated with mitochondrial energetics (24). Furthermore, nHb, at normal levels, acts as a sensor of the energy status of neurons. In linking the ATP concentration and mitochondrial function to mTOR activity, nHb regulates neurochemical stress responses, autophagy, epigenetic changes, and the neurotransmission of dopamine cells (25). In addition, Hbb overexpression increases the level of H3K4me3, which is a histone marker that regulates the expression of oxidative phosphorylation genes (24), which is in accordance with the finding that nHb can regulate the epigenome of neurons upon exposure to stress (25). Overall, it is now clearly established that Hb is expressed locally throughout the brain, where it acts as an oxygen storage entity and is involved in mitochondrial function.

### Growth Hormone—Introduction

GH is a 191-amino acid, single-chain polypeptide. It is synthesized by and secreted from somatotropic cells of the pituitary gland and stimulates cell growth, reproduction, and regeneration (26). It is well-established that GH promotes postnatal growth and metabolism (for reviews, see (26–28). The secretion of GH is regulated by the balanced release of the two hypothalamic peptides: growth hormone-releasing hormone (GHRH) and growth hormone-inhibiting hormone (GHIH or somatostatin). These, in turn, are influenced by many physiologic stimulators (e.g., physical exercise, nutrition, sleep) and inhibitors (e.g., free fatty acids) (29).

GH can act directly on tissues, although many of its effects are mediated by the downstream insulin-like growth factor-I (IGF-I), which is a 70-amino acid polypeptide hormone that is synthesized primarily in the liver (30, 31). Furthermore, circulating IGF-I affects the levels of GH through the classic negative feedback loop formed by the hypothalamic-pituitary axis and the liver (26). The secretion levels of GH and IGF-I are highest during adolescence and decline thereafter in an age-related manner (32).

**Figure 2:**
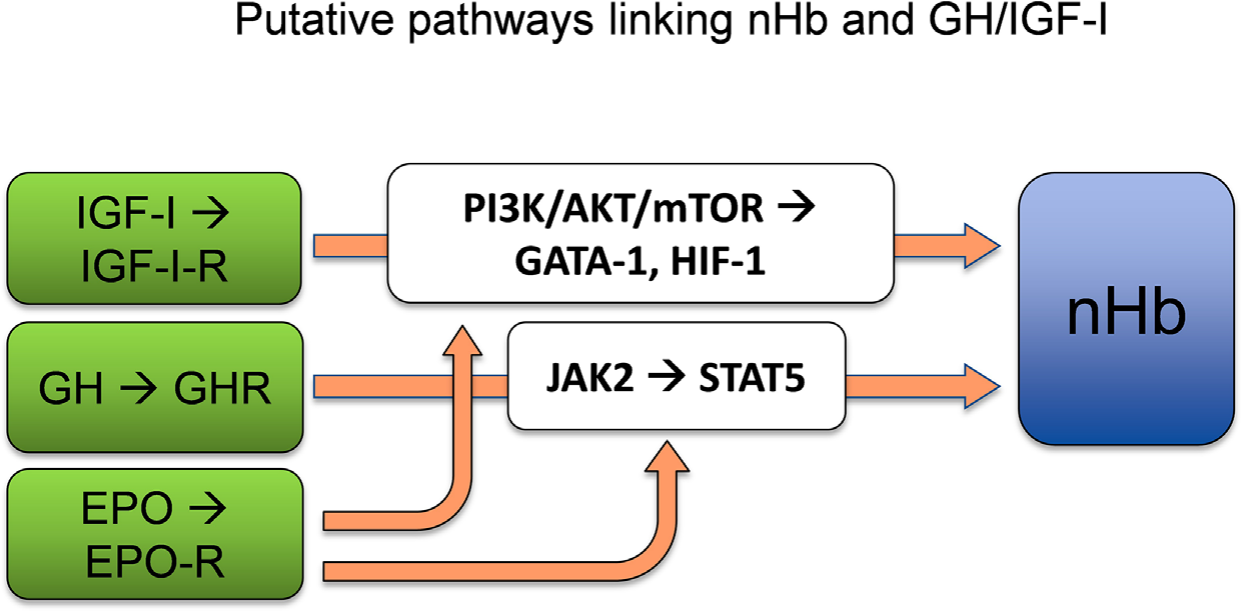
GH and/or IGF-I act as significant determinants of Hb concentrations by adapting adequate levels of Hb to the oxygen demand. STAT5, which is a key transcription factor of GH, is sufficient to allow erythropoiesis and myelopoiesis in vitro and in vivo. The PI3K/AKT/mTOR pathways lie downstream of the IGF-I-receptor. AKT phosphorylation of GATA-1 and its partner FOG-1 coordinates erythroid proliferation and differentiation. HIF-1 induced by IGF-I regulates the supply of oxygen to the cell by creating a balance between oxygen demand and oxygen supply. nHb, Neuronal hemoglobin; GH, growth hormone; IGF-I, insulin-like growth factor 1; STAT5, signal transducer and activator of transcription 5; PI3K/AKT/mTOR, phosphoinositide 3-kinases/protein kinase B/mammalian target of rapamycin; GATA-1, globin transcription factor 1; HIF-1, hypoxia inducible factor 1; FOG-1, friend-of-GATA1. References for the depicted relationships are given in main text.

GH exerts its actions *via* the GH receptor (GHR) (33), which is expressed in virtually every tissue of the body, including the brain (34). From the circulation, GH crosses the blood–brain barrier (BBB) (35, 36), mostly through a process of passive diffusion (37), and binds to the GHR, which is expressed by both neurons and glial cells (38). The dimerization of GHR, which belongs to the type I cytokine receptor family, results in activation of the associated Janus kinase 2 (JAK2) and Src family kinases (34). The activation of JAK2 initiates tyrosine phosphorylation of the signal transducer and activator of transcription 5 (STAT5), the key transcription factor for GH (39). This results in the activation or repression of multiple genes (40), including the stimulation of IGF-I transcription in the liver (30). The EPO receptor (EPO-R) shares homology with the GHR, similarly leading to activation of the JAK2/STAT5 signal transduction pathway (41). It has also been shown that GH induces STAT5 expression in neurons (42), which is of interest with respect to Hb synthesis, as STAT5 activation independently drives erythropoiesis and myelopoiesis, both *in vitro* and *in vivo*, in the absence of EPO-signaling *via* EPO-R or JAK2 (43). Furthermore, experiments have revealed positive correlations between JAK2/STAT5 activation and Hb expression (44). Therefore, STAT5 appears to be a plausible link between GH and the synthesis of the heme portions of Hb, which is known to take place in mitochondria (45). In conclusion, GH appears to act through its key transcription factor STAT5 to enhance nHb expression, although direct evidence for this process is still lacking (**Figure 2**).

### Insulin-Like Growth Factor I—Introduction

IGF-I acts through the IGF-I receptor, which is a hetero-tetrameric glycoprotein that belongs to the tyrosine kinase receptor family. Downstream of the receptor, the activation is mediated by its canonical signaling pathways, such as the PI3K/AKT/mTOR and RAS-RAF-MAP pathways (46). Apart from GH-induced IGF-I production in the liver, which constitutes 75% of the circulating IGF-I (47), IGF-I is also produced locally in tissues, such as the rib growth plate, skeletal and heart muscles (48, 49), and the brain (50).

Both circulating and locally expressed IGF-I affect various brain functions, probably with overlapping and complementary activities in many respects. Nevertheless, circulating IGF-I is believed to mediate some of the effects of circulating GH on the brain [for a review, see (51)]. One line of evidence for this is the robust increase in IGF-I mRNA and protein levels in several major brain regions early (8 h) after systemic GH administration (35, 36). This is possible because IGF-I crosses the BBB *via* at least three different transport systems: 1) carrier-mediated uptake through the endothelial walls (52); 2) the classic endocytic receptor low-density lipoprotein receptor-related protein 1 (LRP1), which can be triggered by neuronal activation (53); and 3) the related receptor megalin/LRP2 in the choroid plexus (54). In most studies, the specific route of IGF-I passage through the BBB is not described, nor investigated. One of the most important actions of circulating IGF-I is neuroprotection. For example, in the brains of rats who have suffered ischemic stroke, IGF-I increases neuronal survival *via* IGF-I receptors (55), thereby improving neurologic functions (46, 56–59).

While circulating IGF-I is important, there are also some indications that locally expressed brain IGF-I is independent of peripheral GH, at least in some situations, such as aging (60) and GH deficiency (GHD) induced by hypophysectomy (61). Local brain IGF-I is subject to complex regulation, which to some extent contrasts with the process of peripheral IGF-I signaling in the body. For example, Ames mice, which have a primary deficiency in relation to GH secretion and therefore have lower levels of circulating GH and IGF-I, still have higher levels of IGF-I in the hippocampus (62). Therefore, although peripheral IGF-I signaling is reduced with aging, brain-specific IGF-I signaling may not necessarily be decreased (63). The opposing effects of peripheral and local brain IGF-I signaling have also been demonstrated with respect to glucose metabolism, as a high level of peripheral IGF-I leads to the accumulation of reactive oxygen species and oxidative stress, while high IGF-I levels in the CNS promote renewal and repair (63).

The IGF-I signaling pathway interacts with the EPO signaling pathway for neuroprotection and Hb regulation (**Figure 2**). Specifically, the IGF-I receptor (IGF-IR) and EPO-R both interact with AKT, which phosphorylates the erythroid-specific transcription factor GATA-1 and its partner FOG-1, which in turn plays a central role in the coordination of erythroid proliferation and differentiation (64). Furthermore, IGF-I acts through the PI3K/AKT/mTOR pathway (65), whereby mTOR plays key roles in identifying nutritional signals, and in promoting cell growth, cell survival, proliferation, and damage repair (65–67). In addition, the PI3K/AKT/mTOR pathway is known to induce the expression of HIF-1α, which is a transcription factor that acts on endothelial cells, among other cell types, and regulates the supply of oxygen to the cell by ensuring a balance between the oxygen demand and oxygen supply (68). Thus, there is considerable support for the notion that IGF-I is involved in Hb regulation, possibly *via* interactions with the PI3K/AKT/mTOR signaling pathways and the transcription factors GATA-1 and HIF-1α.

### GH/IGF-I and Circulating Hb

It is well established that the level of circulating Hb is regulated mainly by EPO [for a review, see (69)]. Moreover, it has been known for quite some time that GH and/or IGF-I, partly independently of EPO, affect to some extent the amount of blood Hb (70–77). For instance, at the end of a 5-year study of GH replacement therapy in children with GHD, the level of Hb was increased and there was also a positive correlation between the serum concentrations of IGF-I and Hb (78). The Hb-increasing effect of GH in children with GHD appears to be specific for erythropoiesis, since it was shown in another report that there were no effects of GH on white blood cells and platelets, regardless of prior history of anemia (79). In analogy, administration to healthy young adult men of a long-acting GHRH analog, which stimulates GH release into the circulation, induced upregulation of the Hbb protein by 0.5–1.0 log units, corresponding to 7–10 Hb units (80). Moreover, GH and/or IGF-I have been reported to associate with Hb in elderly subjects (81). The various reports of increased Hb following GH administration can be explained by direct stimulatory effects of GH and IGF-I on erythroid cells, in combination with a more generalized indirect effect of GH in facilitating physical activity through increased muscle performance and well-being (82), which in turn enhances general health and Hb production. Conceptually, this may be mediated by the general anabolic effect of GH, which is considered to stimulate protein and fat metabolism, which in turn requires increases in oxygen transportation and Hb levels (83).

IGF-I has also been shown to stimulate erythropoiesis *in vivo* independently of GH, which means that it could indirectly mediate GH effects on EPO synthesis, erythropoiesis, and Hb regulation. As compared to EPO, which has a substantial and significant effect on Hb levels, the effect of serum IGF-I has been shown to be less-prominent than but still independent of circulating EPO (84). Moreover, it has been suggested that IGF-I is a factor that coordinates red blood cell formation with organ and body growth, and this would account for the adaptation of red blood cell mass to whole body mass (84). From another publication, it can be calculated that an increase of 100 units of serum IGF-I (ng/ml) is associated with an increase of 4.0 units of Hb (g/L) in males and 7.5 units in females (81). A difference in serum IGF-I of 100 ng/ml is rather large considering that the mean serum IGF-I level in middle-aged men and women is in the range of 150–200 ng/ml. The serum IGF-I is also about 100–200 ng/ml higher in young adults than in middle-aged subjects (85). Taken together, these findings indicate definite albeit moderate effects of endocrine GH and IGF-I on circulating Hb.

### GH/IGF-I and CNS Expression of Hb

Given that endocrine GH and IGF-I stimulate circulating Hb, it is conceivable that they also affect local brain expression of Hb, although the quantity and nature of the expressed induced Hb might be different. In recent years, this possibility has been examined from several perspectives in a number of experimental studies. For example, GH replacement in the GH/IGF-I deficient (Lewis dwarf [dw/dw]) rat robustly increased the transcription levels of Alox15, Hba, and Hbb in the hippocampus (86). The authors of that study imply that this indicates a mechanism through which IGF-I regulates vascular function by decreasing oxidative stress in the brain (87), and they suggest that these effects are mediated by Hb and other globins (86). The notion that GH and/or IGF-I act on brain Hbb is supported by our experiments, in which we observed a substantial decrease in the hippocampal and cortical levels of the *Hbb* transcript when comparing hypophysectomised (GH-deficient) rats with intact rats, and this effect was noted in both female and male animals (88, 89). Furthermore, GH administration for 6 days to hypophysectomised female rats (90, 91) and male rats (88) robustly (2–4-fold) upregulated the number of *Hbb* transcripts in the brain. Furthermore, GH administration to the rats for 19 days increased the mRNA levels of *Hbb*, *Alas2*, and *Alox15.* For *Hbb* and *Alas2*, the effect size was of a magnitude similar to that seen after 6 days of GH treatment, while *Alox15* was not studied in the 6-day experiments (89). Taken together, the different GH administration regimens indicate that the GH effect is not markedly diminished or enhanced over time of administration. For humans, it has also been observed that the level of serum Hbb decreases following pituitary surgery (92). However, the regulation of *Alas2* and *Alox15* by GH presents a new target of action for GH, since they both have functional links to aerobic metabolism. Specifically, Alas2, which is expressed in the mitochondria of bone marrow cells and red blood cells, and to some degree in the brain (https://www.genecards.org, [Accessed May 26, 2020]), has been shown to be increased by oxygenation of the tissue (93). Alox15, which is expressed in the mitochondria of lung cells and adipocytes, as well as in the brain (https://www.genecards.org, [Accessed May 26, 2020]), has been reported to regulate vascular tone, local blood flow, and blood pressure (94). In addition to the GH administration experiments, we have shown that IGF-I-treatment can increase the expression levels of *Hbb*, *Alas2*, and *Alox15* (91).

The effects of GH and IGF-I on local brain Hbb expression may be analogous to the endocrine effects on general metabolism requirements, which in turn affect the oxygen demand, and thereby the rate of erythropoiesis (95). Based on these findings, the basal metabolic rate may be lowered after hypophysectomy, with GH restoring the basal metabolism and oxygen consumption, thereby affecting the neuronal oxygen-binding capacity and the levels of vascular-associated transcripts (89, 96). This idea is supported by a study showing that bovine GH-transgenic mice exhibit an increased basal metabolic rate (97). In summary, there is evidence that both GH and IGF-I, independently of EPO, can augment the level of Hb both systemically and locally in the CNS, especially in the case of GH deficiency.

## GH/IGF-I and Various Brain Diseases in Relation to Hb

### Stroke and Hemoglobin

Hb is important in relation to brain injuries in several aspects. The most common brain injuries are ischemic stroke, hemorrhagic stroke, and perinatal asphyxia. Ischemic strokes, regardless of etiology, result in an inadequate blood supply that leads to cell death within the affected tissue [for reviews, see (98–100)]. Ischemic pre- or post-conditioning is an experimental stroke paradigm that can reduce the ischemic injury. Interestingly, ischemic preconditioning to hypoxia *in vitro* robustly increases nHb expression in neuronal cultures from E-17 rats (101). A plausible explanation for this is that Hb inhibits oxidative stress-induced mitochondrial dysfunction and caspase activation *via* the protein kinase A, protein kinase C, and mitogen-activated protein (MAP)-kinase signal transduction pathways (102).

In contrast to the intracellular expression of Hb, extracellular Hb is highly toxic to the brain and constitutes a mechanism of damage after ischemic stroke, and more profoundly so after hemorrhagic stroke (103). Specifically, free Hb induces the widespread and concentration-dependent death of neocortical neurons *in vitro* (104, 105). Similarly, intracerebral injections of free Hb or its degradation products, including heme, induce brain injury *in vivo* (106). While the exact mechanisms are unknown, it appears that extracellular Hb contributes to oxidative stress and iron accumulation in neurons, resulting in a deleterious cycle of heme breakdown, Hb denaturation, and, over time, neurodegeneration (107). Overall, it seems that extracellular free Hb activates deleterious cellular pathways, whereas intracellular Hb is primarily protective (20).

In addition, circulating Hb may affect stroke risk, severity, and outcome, presumably through mechanisms other than the neuronal expression of Hb. For example, circulating Hb seems to influence stroke risk, since anemia increases the risk of stroke and worsens stroke outcomes (108). Moreover, in acute ischemic stroke, low levels of circulating Hb independently predict short- and long-term mortality (109), correlate inversely with larger stroke volumes (110), and are strongly associated with subsequent decreases in the Hb and hematocrit levels (111). Interestingly, in comparison to anemia, polycytemia with high levels of Hb (≥15 g/L in men, ≥14 g/L in women) has been associated with twice as many cerebral infarctions (112, 113). This is thought to be mediated by increased viscosity of the blood and comorbidities, causing an increased risk of thrombosis (113). Furthermore, once the clot has been formed, it starts to resolve, which in turn causes erythrocyte lysis and the release of free Hb, which leads to an overload of free, toxic iron (103).

Although several studies have shown that GH and IGF-I either protect against injury or promote recovery, these effects have not so far been linked to Hb regulation. It has been shown that GH protects the brain against hypoxic-ischemic injuries (HI) in neonatal rodents (114) and increases motor and cognitive recovery in adult rodents (115–117). Furthermore, the positive effects on functional recovery of GH treatment also appear to be present in humans who are suffering from chronic stroke, as shown by the increased amplitude of blood oxygen level-dependent signals in the brain, which are dependent upon the different magnetic properties of oxy-Hb and deoxy-Hb (118). That the effects of systemic GH treatment are mediated by local or endocrine IGF-I with respect to injuries is not clearly elucidated but certainly possible, as the IGF-I system in the brain shows marked changes in response to transient neural injuries. For instance, in asphyxia, there is an increase in the level of *Igf1* in glial cells in the region of the injury (119), suggesting that the IGF-I system is involved in neuroprotection. IGF-I administration has been shown to be both neuroprotective (120) and recovery-promoting (121) in studies of experimental ischemic stroke [for a review, see (59)] and the articles (116, 117, 122). However, the role of circulating IGF-I and its associations with functional outcome in human stroke appear to be complex. While high serum levels of IGF-I have been associated with both worse outcomes (123, 124) and greater stroke severities, they are also linked to better long-term recovery (85, 125). These discrepancies can partially be explained or confounded by the fact that the levels of serum IGF-I are lower in aged, malnourished, and/or sedentary subjects (71) and may depend on the post-stroke sampling time-point, as suggested previously (122). Although the absolute levels of serum IGF-I appear to predict functional outcome, post-stroke dynamic changes (decreases) in the levels of serum IGF-I may also be at least as important (122, 126). Some studies have shown a weak-to-moderate correlation between serum IGF-I and Hb (64, 65, 68), a relationship that has not been investigated in other studies (85, 122, 125). That the functional outcome of stroke seems to be dependent upon the status of the GH/IGF-I axis indicates that there is a correlation with Hb, and this aspect deserves further investigation.

### Traumatic Brain Injury

Traumatic brain injury (TBI), which is a risk factor for depression, cognitive impairment, and hypopituitarism, entails decreased levels of circulating GH and IGF-I and has links to circulating and brain Hb. For example, among patients with severe TBI, lower levels of Hb are consistently associated with lower survival rates (127) and poor outcomes (128). While anemia frequently occurs after TBI, the associations with outcome and the effects of blood transfusions are rather inconsistent [for a review, see (129)]. Moreover, studies of post-TBI neuroendocrine dysfunction have found that the somatotropic axis (GH and IGF-I) seems to be the most commonly disrupted pituitary hormonal axis, in similarity to prolactin hormone disruption, whereas the thyrotrophic, corticotrophic, and gonadotrophic axes are less-affected (130), suggesting that GHD is linked to anemia. Interestingly, GH replacement has a positive effect on cardiorespiratory fitness in individuals with TBI (131). GH therapy also ameliorates neuropsychological and psychiatric changes 3 years post-TBI (132), and improves disabilities and cognitive impairments in patients with TBI (133). In animal studies, transgenic mice with astrocyte-specific overexpression of IGF-I have exhibited increased neuroprotection against TBI-induced injury in the hippocampus, resulting in reduced motor and cognitive dysfunctions (134). As mentioned above, since IGF-1 may mediate the effects of GH, both GH (or IGF-I) administration paradigms, including acute and long-term treatments, may have significance for TBI outcomes and the Hb levels in the brain. Thus, there are indications that altered Hb levels are part of the recovery response to GH treatment, a topic that warrants exploration in future studies.

## Degenerative Brain Diseases, Hb, and GH/IGF-I

Alterations in cerebral oxygen metabolism have been found to be associated with many of the most common degenerative brain disorders, such as Alzheimer’s disease (AD), Parkinson’s disease (PD), and multiple sclerosis (MS), and the CNS expression of Hbb has been implicated in these diseases (135).

### Alzheimer’s Disease

AD is the most common cause of dementia, and the risk of AD is higher when there is a history of brain lesions, such as TBI (136, 137) or stroke [for review, see (138)]. Early clinical symptoms include difficulties in remembering recent events and conversations, and ultimately there are reduced abilities to walk, speak and swallow. The histopathologic hallmarks of AD are the progressive accumulation of beta-amyloid (Aβ) peptide plaques outside neurons in the brain, twisted strands (tangles) of tau protein inside neurons (139), and a general hyperphosphorylation of the tau protein (140). Eventually, these changes result in damage to and death of neurons [Alzheimer’s disease facts and figures (141)].

As is the case for AD and brain Hb expression, several studies have indicated a role for Hb in AD. In humans, Hb protein has been detected in the neurons and glial cells of post-mortem AD brains. Furthermore, the level of local Hb protein has been found to be increased in AD brains (142). This indicates that either the upregulation of Hb expression or leakage of Hb from the circulation into the brain parenchyma (due to disruption of the BBB) is involved in AD pathogenesis (142). In animals, Hbb is upregulated with normal aging, as well as in a amyloid precursor protein/PS1 transgenic mice (murine model of AD), possibly serving as a compensatory mechanism for hypoxia (143). In addition, when micro-hemorrhaging occurs as the result of aging, TBI or cerebrovascular disease, Hb from red blood cells can bind to the Aβ peptide *via* the iron-containing heme moiety and accelerate its aggregation (143).

Furthermore, altered iron metabolism has been observed in the CNS of patients with a variety of neurodegenerative diseases, including AD. The concomitant occurrence of a decrease in local Hb expression in AD and the increased level of free iron, which is highly toxic due to the creation of reactive oxygen species (144), is possibly coincidental, although it indicates the importance of iron metabolism. Epidemiologic studies have suggested strong associations between anemia, decline of cognitive function, and AD, with an inverse relationship noted between Hb and AD (145–147). Moreover, in a large population-based study, it has been shown that low Hb levels are associated with increased long-term risks of dementia (34%) and AD (41%) (148).

Aβ activates a molecular response pathway that leads to strong downregulation of Hb gene expression in the brain, which could damage cells due to defective oxygen homeostasis (144). Reduced concentrations of the Hb α-chain and β-chain proteins have been detected in neurons with granular or punctuate hyperphosphorylated tau deposits, in AD neurons with tangles in the hippocampus and frontal cortex, and in AD neurons in the amygdala (135). These findings are in line with a report showing significantly lower levels of mean oxygen metabolism in the AD cortex (149). Loss of Hb is specific, as related transcripts, i.e., for Ngb and the EPO receptor, are expressed at normal levels (135).

There are strong indications that IGF-I is of importance in the development and progression of AD. A deficiency of liver-derived circulating IGF-I in mice resulted in debilitated spatial learning and memory (150), as well as reduced exploratory activity (151). In experimental AD, the passage of IGF-I through the BBB has been shown to be reduced (152), while systemic IGF-I infusions increased Aβ clearance, thereby reducing the brain level of Aβ (152). In a later study, a specific BBB disruption allowing serum IGF-I entrance into the rodent brain was demonstrated, as the choroid plexus receptor megalin/LRP2 induced IGF-I transport across the BBB and protected against AD, and in AD mice, megalin abundance was decreased (54). Furthermore, IGF-I not only interacts with Aβ metabolism and distribution, but also with tau metabolism, as evidenced by the finding that the phosphorylation of tau is markedly increased in the IGF-I-null brain in mice (153).

In a prospective, population-based study of older persons, low IGF-I status (<9.4 nmol/L) was associated with both lower levels of cognitive ability and a sharper decline in cognition (154). In a case-control study, lower serum IGF-I levels were significantly related to dementia that was clinically diagnosed as AD (155). However, discrepant results have been reported with respect to the serum and cerebrospinal fluid (CSF) levels of IGF-I in patients with AD. It has been postulated that these discrepancies can be explained in terms of resistance to IGF-I action early in AD development (reflected as increased serum IGF-I), followed by IGF-I deficiency when the disease progresses (reflected as lower serum IGF-I) (156). Resistance to IGF-I signaling early in AD could result in a lack of trophic signals, with subsequent degeneration of neurons (157). Post-mortem studies have shown reduced IGF-I gene expression in AD brains (158, 159), and this was accompanied by resistance to signaling through the IGF-I receptor (158, 159). Talbot et al. have confirmed the presence of resistance to IGF-I receptor signaling by demonstrating that in the post-mortem AD brain, the signaling responses to IGF-1 in the IGF-IR/PI3K/AKT/mTOR pathway are strongly attenuated (160). This dysfunction may result in abnormal responses to mTOR activation in the brain, leading to an AD-type pathology, neuronal death, and glial activation (67).

In human AD, in similarity to the previous experimental findings (54, 152), there also appears to be reduced passage of IGF-I through the BBB, as indicated by the lower CSF/serum IGF-I ratios detected in patients with AD (161, 162). Finally, in a recent study of patients with AD, the serum level of IGF-I was associated with the CSF level of Aβ, whereas the CSF level of IGF-I was associated with the CSF level of tau (163). These findings are in accordance with the findings in rodents that IGF-I in the serum has the ability to interact with Aβ clearance, possibly, at least partly, at the level of the BBB (54, 152), whereas IGF-I in the brain has the ability to affect the intracellular metabolism of tau (153).

In summary, there is considerable support for the notion that anemia is a risk factor for dementia, including AD, and that leakage of Hb through a defective BBB can result in an overload of toxic iron in patients with AD. Furthermore, in AD, alterations to both the levels and activity of IGF-I have been documented. However, the pathways that connect IGF-I and local Hb have not been explored in detail. Clearly, more research is needed to elucidate whether the pathologic alterations in the Hb and IGF-I systems interact in AD, resulting in aggravated development and accelerated progression of the disease. PI3K/AKT/mTOR signaling could be one of the molecular pathways that link IGF-I and Hb.

### Parkinson’s Disease

PD primarily affects movement coordination, with symptoms such as slowness, rigidity, tremor, and changes in gait. Neurobiologically, PD is characterized by chronic and progressive degeneration of dopamine-producing neural cells in the substantia nigra, which are essential for the activation of a circuit that controls movement [for a review, see (164)].

With respect to PD, a key molecule is brain α-synuclein, which is present at high levels and is concentrated in the presynaptic nerve terminals. It is involved in controlling the plasticity of dopamine overflow, synaptic vesicle recycling, storage, and the compartmentalization of neurotransmitters (165). In PD, α-synuclein aggregates into Lewy bodies, the abnormal intracellular inclusions that are the classical pathologic hallmark of the disease (166). As PD progresses, it often results in dementia secondary to the accumulation of Lewy bodies in the cortex or the accumulation of Aβ clumps and tau tangles, not unlike the situation in AD [Alzheimer’s disease facts and figures (141)]. It can also be mentioned that PD dementia is closely related to Lewy body dementia, with the clinical features of the latter mainly differing in that its first clinical feature is dementia, with possible later motor impairments [for a review, see (167)]. Also, symptoms like those of PD, verified with neurobehavioral scores, may be observed after carbon monoxide intoxication which binds to hemoglobin instead of oxygen, possibly acting in a degenerative way (168). Bearing in mind that nHb plays a vital role in maintaining normal mitochondrial functionality in the brain, it is interesting that Hb is normally highly expressed in the A9 dopamine neurons of the substantia nigra (135, 169). However, in about 80% of neurons with punctuate α-synuclein deposits and in neurons with Lewy bodies in the substantia nigra and the medulla oblongata, there are clear reductions in the concentrations of Hb α-chain and β-chain proteins (135). Furthermore, mitochondrial Hb protein levels decrease with age in the striatum as a result of increased intracellular α-synuclein accumulation and formation of Hb-α-synuclein complexes, which may contribute to α-synuclein-induced mitochondrial dysfunction and increase the risk of PD (170). This results in a reduced translocation of nHb from the cytoplasm to the mitochondria which may contribute to mitochondrial dysfunction and thereby accelerating PD pathogenesis (170, 171). This is in line with a proposed hypothesis, stating that the oxidative damage contributes to the neurodegenerative process in PD *via* nHB per se (169). Thus, it seems that there are relatively strong links between PD and Hb regulation in the brain.

However, the molecular pathways linking local Hb with IGF-I in cases of PD have been scarcely explored, despite there being indirect indications of possible pathways from other studies of IGF-I. The potential involvement of IGF-I is indicated by the fact that A9 dopamine neurons specifically express GATA protein family members, which are the major Hb transcriptional regulators (22, 172), controlled by the IGF-IR through the AKT-pathway (64). In turn, α-synuclein is an essential regulator of IGF-I-mediated AKT activation, and by binding to the kinase domain of AKT it protects against the aggregation of AKT (173). However, when α-synuclein is overexpressed, activated or inactivated, AKT is sequestered and the IGF-I-mediated survival signal is not propagated (173). Moreover, examination of the clinical correlations in PD cohorts has confirmed that the levels of serum IGF-I is increased at PD onset, suggesting a compensatory mechanism. Furthermore, in women, estrogens seem to confer strong protection against PD *via* serum and CSF IGF-I (174). In addition, erythropoietin has been shown to have a potential neuroprotective role in the maintenance of dopaminergic neurons in a rat model of PD (175).

PD may also be linked to iron deficiency, since overexpression of Hbb in dopamine cell lines alters transcription related to iron metabolism, O_2_ homeostasis, oxidative phosphorylation, and nitric oxide synthesis (22). Interestingly, both *Hbb* and *Alas2* are upregulated in iron-deficient mice (176), and these were the two most strongly upregulated transcripts in our experiments involving GH and IGF-I supplementation (88, 89). In addition, free ferrous (Fe^2+^) and ferric (Fe^3+^) iron species are cytotoxic and have been suggested to enhance oxidative stress, mitochondrial protein dysfunction, α-synuclein aggregation, neuronal cell death (107, 177), and lipid peroxidation (178).

Overall, reduced local expression of Hb, specifically in mitochondria, together with an altered iron metabolism and the mutual influences of IGF-I and α-synuclein on AKT regulation may have implications for the development of PD. However, further investigations are needed.

### Multiple Sclerosis

MS is primarily an inflammatory disorder of the brain and spinal cord, in which focal lymphocytic infiltration leads to damage to myelin and axons. Initially, the inflammation is transient and re-myelination occurs, but this most often does not persist and the result is chronic neurodegeneration and progressive disability [for a review, see (179)]. Interestingly, there are some lines of evidence to suggest a link to Hb expression in the CNS.

In a study of eight monozygotic twin pairs discordant for MS (180), >2-fold upregulation of six gene transcripts was detected in mononuclear cells isolated from the peripheral blood, amongst them *Hba2* and *Hbb*, suggesting some role for these genes in MS pathogenesis (180). This is in line with a report showing increased expression of local Hbb protein in the cortex, as well as intense Hb staining in the pyramidal neuronal cell bodies of patients with MS (181).

Specifically, in MS, it has been suggested that neuronal Hbb is part of a mechanism linking neuronal energetics with epigenetic changes in the nucleus, providing neuroprotection by supporting neuronal metabolism (24). Dysregulation of Hbb transport into the nuclei of pyramidal neurons in MS has been suggested by the findings that in patients with MS/experimental MS there is a reduction of the histone marker H3K4me3 and downregulation of genes involved in mitochondrial respiration (24). In contrast, free Hb originating from dying erythrocytes is detrimental in MS pathogenesis, as it causes damage to the myelin and BBB (182). In addition, an association between anemia and MS has been described (183).

There are also indications that the GH/IGF-I system is involved in MS. First, the levels of CSF-GH were found to be 2-fold lower in patients with MS (184). In contrast to CSF levels of GH, the serum levels of GH were unchanged, which suggests normal pituitary function and, instead, indicates malfunction of the BBB or in the local production of GH in the brain. The levels of IGF-I in serum and CSF also showed a trend toward divergence (184). Furthermore, MS and higher levels of disability seem to be associated with a reduction in the bioavailability of IGF-I, possibly due to high serum concentrations of IGF-binding protein-3 (185). Taken together, these findings suggest that Hbb dysregulation and GH abnormalities are present in MS, although to our knowledge, comprehensive evidence for a direct linkage between the two factors is still lacking.

## Summary

In summary, GH and IGF-I are significant determinants of Hb concentrations, adapting the local nHb and circulating HbA levels to the demand for oxygen. Both local nHb expression in the brain and GH/IGF-I levels influence cardiovascular and neurodegenerative diseases, such as ischemic injury, TBI, AD, PD and MS. There are indications that the connection between GH and Hb may be related to STAT5, and for IGF-I and Hb, this linkage is likely mediated by the PI3K/AKT/mTOR pathway. It will be of interest to investigate further the signaling pathways linking GH/IGF-I to Hbb and their relationships to neuroprotection. Hopefully, this will lead to improved therapeutic strategies to stimulate recovery in patients after brain injuries, e.g., after stroke, TBI, and neurodegenerative diseases.

## Author Contributions

MW wrote the first draft of the manuscript. DÅ contributed significantly to the second version. MW, JS, LK, MÅ, RM, GK, JI, and DÅ contributed to manuscript revision, read, and approved the submitted version. All authors contributed to the article and approved the submitted version.

## Funding

This study was supported by the Swedish Medical Society (*Svenska Läkaresällskapet*), grants from the Swedish State under the agreement between the Swedish Government and the County Councils (ALFGBG-719761 and ALFGBG-722371), the Swedish Stroke Association, and Stiftelsen Peter Erikssons Minnesfond.

## Conflict of Interest

The authors declare that the research was conducted in the absence of any commercial or financial relationships that could be construed as a potential conflict of interest.
